# Modulation of Structure and Optical Property of Nitrogen-Incorporated VO_2_ (M1) Thin Films by Polyvinyl Pyrrolidone

**DOI:** 10.3390/ma16010208

**Published:** 2022-12-26

**Authors:** Meinan Wan, Mo Xiong, Shouqin Tian, Xingzhu Chen, Bin Li, Xuesong Lu, Xiujian Zhao

**Affiliations:** 1 School of Architectural Engineering, Huanggang Normal University, Huanggang 438000, China; 2 MOE Key Laboratory for Non-Equilibrium Synthesis and Modulation of Condensed Matter, School of Physics, Xi’an Jiaotong University, Xi’an 710049, China; 3 State Key Laboratory of Silicate Materials for Architectures, Wuhan University of Technology, Wuhan 430070, China; 4 KAUST Catalysis Center, King Abdullah University of Science and Technology, Thuwal 23955, Saudi Arabia; 5 Huanggang Ecological Architecture and Renewable Resources Research Center, Huanggang 438000, China

**Keywords:** VO_2_ (M1) thin films, nitrogen-incorporated, polymer-assisted sol–gel method, optical property, polyvinyl pyrrolidone (PVP), first-principles calculations

## Abstract

VO_2_, as a promising material for smart windows, has attracted much attention, and researchers have been continuously striving to optimize the performance of VO_2_-based materials. Herein, nitrogen-incorporated VO_2_ (M1) thin films, using a polyvinylpyrrolidone (PVP)-assisted sol–gel method followed by heat treatment in NH_3_ atmosphere, were synthesized, which exhibited a good solar modulation efficiency (*ΔT_sol_*) of 4.99% and modulation efficiency of 37.6% at 2000 nm (*ΔT_2000 nm_*), while their visible integrated transmittance (*T_lum_*) ranged from 52.19% to 56.79% after the phase transition. The crystallization, microstructure, and thickness of the film could be regulated by varying PVP concentrations. XPS results showed that, in addition to the NH_3_ atmosphere-N doped into VO_2_ lattice, the pyrrolidone-N introduced N-containing groups with N–N, N–O, or N–H bonds into the vicinity of the surface or void of the film in the form of molecular adsorption or atom (N, O, and H) filling. According to the Tauc plot, the estimated bandgap of N-incorporated VO_2_ thin films related to metal-to-insulator transition (*E_g_*_1_) was 0.16–0.26 eV, while that associated with the visible transparency (*E_g_*_2_) was 1.31–1.45 eV. The calculated *E_g_*_1_ and *E_g_*_2_ from the first-principles theory were 0.1–0.5 eV and 1.4–1.6 eV, respectively. The Tauc plot estimation and theoretical calculations suggested that the combined effect of N-doping and N-adsorption with the extra atom (H, N, and O) decreased the critical temperature (*τ_c_*) due to the reduction in *E_g_*_1_.

## 1. Introduction

Monoclinic phase vanadium dioxide [VO_2_ (M1)] has attracted great attention for smart coating applications because of its promising thermochromic properties [1,2,3,4]. VO_2_ (M1) material undergoes a reversible metal-to-insulator transition (MIT) at a critical temperature (*τ_c_*) of around 65 °C. It exhibits an insulating monoclinic (M1) phase at room temperature and exhibits metal characteristics as a rutile (R) phase while above the critical temperature. However, the high phase transition temperature (*τ_c_*), low luminous transmittance (*T_lum_*), not remarkable enough thermochromic modulation efficiency for solar energy (*ΔT_sol_*) or near-infrared modulation efficiency (*ΔT_NIR_*), and the less desirable color of the intrinsic VO_2_ (M1) thin films constrain the practical application of VO_2_ materials. The thermochromic performance of VO_2_ is closely related to the phase composition and the microstructure, which are largely dependent on the synthesis method and growth control. Furthermore, changing the microstructure and the thickness of the VO_2_ (M1) thin film can significantly regulate its physical properties.

Polymer-assisted deposition (PAD) [5,6,7] is a chemical solution route to high-quality thin films of metal oxides. This technique employs coordination between metal ions and polymers to guarantee the homogeneous distribution of metal ions and, thus, the formation of uniform VO_2_ thin films. It provides a cost-effective and scalable alternative method for the sol–gel method. Polyvinylpyrrolidone (PVP) is a synthetic water-soluble polymer compound that is soluble in both water and most organic solvents. PVP is widely used due to its colloidal protection, film-forming and bonding effects, and performance advantages of very low toxicity and good physiological compatibility. Kang et al. [6] revealed that PVP not only acted as a film-forming promoter but also facilitated the formation of M1/R-phase VO_2_. PVP could promote the formation of crosslinked high-quality gel films after solvent evaporation via the interactions among the charged amide groups and metal ions. At the same time, PVP could also prevent the segregation of inorganic solute in the precursor solution through the interaction between the polymer and the metal ions. Furthermore, VO_2_ film prepared using the PAD method through spin-coating is not as compact as that fabricated using physical vapor deposition methods [8,9,10]. This is beneficial to the improvement of visible-light transmission.

Element doping can also effectively adjust the performances of VO_2_ at the electronic structure level, as the *τ_c_*, the *T_lum_*, or the film color can be modulated by changing the MIT-related band gap (*E_g_*_1_) or visible-transparency-related band gap (*E_g_*_2_). The metal elements used for doping in the VO_2_ films include W [11,12,13], Ta [14], Nb [15], Cr [16], Tb [17], Mo [18], and Mg [19,20]. A dopant with high valence leads to an n-type conductivity, which reduces τ_c_. Doping with Mg improves visible-light transmission and increases *E_g_*_2_, which lightens the yellow appearance of VO_2_ films. Doping with nonmetal elements such as H [21,22], B [23], F [24], Ar [25], and N [8,26,27] can also modulate the *τ_c_* of VO_2_ (M1). For example, N-doped VO_2_ thin films prepared by low-energy N_2_^+^ ion sputtering and annealing in an ultrahigh-vacuum chamber exhibited a reduction in electrical *τ_c_* by 18 °C [26]. Another N-doped VO_2_ (M1) thin film deposited on fused silica substrate at 500 °C by reactive pulsed laser deposition (RPLD) using a VN target reactive with O_2_ flow demonstrated a decrease in optical *τ_c_* by 16 °C extracted from infrared reflectance at λ = 12 μm [8]. Moreover, N-doped VO_2_ (M1) films can be obtained via the chemical solution route using a heat treatment in a vacuum environment with trace NH_3_ concentration, showing an optical *τ_c_* by 17 °C estimated from the thermal hysteresis loop of transmittance measured at λ = 2 μm [27]. Despite the different methods of preparation and phase transition temperature testing of N-doped VO_2_, all cases revealed that N doping can lower the *τ_c_* by 16–18 °C.

As mentioned above, N doping can effectively modulate the optical and electrical properties of the VO_2_ thin films. In our previous work, it was reported and confirmed that annealing in an NH_3_ atmosphere is a facile way to fabricate N-doped VO_2_ (M1) thin films [27,28]. Nitrogen element can be doped into the VO_2_ lattice but exists in two different forms: substitutional N-doping and interstitial N-doping. However, as the VOCl_2_-PVP sol precursor contains pyrrolidone-N from PVP, this N source may also introduce nitrogen elements into the VO_2_ thin film. Following annealing in the NH_3_ atmosphere, the final forms of N cooperating with other elements might also affect the properties of VO_2_ thin films, which has not been discussed so far.

In this work, we fabricate VO_2_ (M1) thin films via spin coating and enclosed NH_3_ atmosphere heat treatment using VOCl_2_-PVP as the precursor. The effects of PVP content in the precursor on the composition, structure, and properties of VO_2_ thin films were studied. The microstructures and optical properties including *T_lum_*, *ΔT_sol_*, *ΔT_NIR_*, modulation ability at 2000 nm (*ΔT_2000 nm_*), and *E_g_* of VO_2_ films were adjusted by varying the concentration of PVP in the precursor. Meanwhile, the possible influences of various forms of N elements including N-doping and N-adsorption introduced in the system on the properties of VO_2_ thin films were analyzed and discussed. According to the XPS analysis results, the N introduced by PVP from the precursor mainly presented in the adsorbed state in the VO_2_ film. Moreover, first-principles calculations were performed to inspect the band structures of VO_2_ affected by different bonding models between N and additional elements (H, N, O, and C).

## 2. Materials and Methods

### 2.1. Sample Preparation

The VO_2_ thin films were synthesized using the polymer-assisted chemical solution method. Vanadium (V) pentoxide (V_2_O_5_, 99.9%,) and hydrazine dihydrochloride (N_2_H_4_·2HCl, 99.9%,) were the raw materials for the preparation of vanadium-containing solutions. PVP (K88-96, Aladdin reagent Co., Ltd., Shanghai, China) was employed as the film-forming promoter. A yellow or orange aqueous suspension (~50 mL) containing 1.820 g of V_2_O_5_ was kept at 60 °C with continuously stirring. Then, the suspension color turned dark to yellowish brown, as 0.525 g of N_2_H_4_·2HCl was added. The suspension color continued to change from dark green to blue through the slow addition of a concentrated HCl solution (38%, 4 mL). Finally, a clear blue solution of VOCl_2_ was formed after heating and stirring for an appropriate period. The concentration of VOCl_2_ solution was then adjusted to 0.1 mol/L, and different contents of PVP (0 wt.%, 3 wt.%, 6 wt.%, and 9 wt.%) were added. Accordingly, the film samples prepared from the VOCl_2_-PVP precursors with different PVP contents were labeled as P1 (or 0%), P2 (or 3%), P3 (or 6%), and P4 (or 9%).

All the precursor films were spin-coated on quartz glass (25 mm × 25 mm) at 500 rpm for 9 s, followed by 3000 rpm for 30 s. Subsequently, the wet sol films were dried at 70 °C for 10 min. The coating and drying procedure was repeated once. The annealing procedure was implemented in a closed vacuum tube furnace. The amount of NH_4_HCO_3_ deposited in the furnace was 0.1 g. Meanwhile, 1 g of CaO powder was also placed to establish a dry NH_3_ atmosphere during heat treatment. The vacuum pump was turned off when the base pressure reached 1800 ± 100 Pa. All film samples were annealed in the furnace at 500 °C with a heating rate of 10 °C/min and then kept at this temperature for 30 min. More details about the preparation of vanadium-containing sol and dry gel film can be found in [27,28].

### 2.2. Characterization

The crystal phase of the thin films was ensured using glance incident X-ray diffraction (GIXRD, Empyrean, Panalytical Inc., Almelo, The Netherlands) at room temperature with a grazing angle of 0.5°. Raman spectra were measured through a Raman microscope spectrometer (Invia, Renishaw, Wotton-under-Edge, UK) using a laser emission wavelength of 632.8 nm with an output power of 5 mW. According to the reproducibility correction of the 520 cm^−1^ peak position of monocrystalline silicon, the error was less than 0.03 cm^−1^. The micro-morphologies of the film samples were observed by field-emission scanning electron microscopy (FESEM, ULTPAPLUS-43-13, Zeiss, Jena, Germany). The FESEM sample preparation method was applied to cut the VO_2_ film-coated quartz glass into small pieces of about 2 × 2 × 1 mm, to glue the back side of the glass (film side up) to the sample table with conductive tape, and to spray carbon before electron microscope observation. The X-ray photoemission spectroscopy (XPS) measurements were recorded using a THERMO PHI Quantum 2000 system. Since V on the surface of the film is easily oxidized to a high valence state, the chemical binding energy of elements inside the sample was studied by sputtering etching with an Ar^+^ ion beam at a depth of about 1 nm/min. The peak positions of elements in the test results were corrected through the 284.6 eV peak of C1s or 530.0 eV peak of O1s using XPSPEAK4.1. Optical performance tests were carried out on a UV/Vis/NIR spectrometer (UV-3600, Shimadzu, Kyoto, Japan) in the wavelength range of 300–2500 nm. Transmittance spectra before and after phase transition were recorded at room temperature (around 20 °C) and heated temperature (set at 90 °C), respectively. The details of the optical performance test and dimming performance evaluation methods of thin films can be found in [27,28].

### 2.3. Ab Initio Calculations

On the basis of the experimental results of XPS, density functional theory (DFT) in the Vienna Ab initio Simulation Package (VASP) [29] was used to ab initio calculate the electronic band structures of various N-containing VO_2_ systems. All structures were completely relaxed and highly accurate. The GGA + *U* method [30] was employed, where the effective Coulomb repulsion potential *U*_eff_ = *U* − *J* was set to 3.4 eV [31,32]. The cutoff energy was set as 600 eV, and a 5 × 5 × 5 Monkhorst–Pack grid of *k* points was selected for all structures. The total energy convergence criterion for electron self-consistent rings was 1 × 10^−5^ eV. The geometry of the system was completely relaxed to a maximum force of less than 0.01 eV/Å.

## 3. Results and Discussion

### 3.1. Phase Determination of VO_2_ (M1) Thin Films

**Figure 1:**
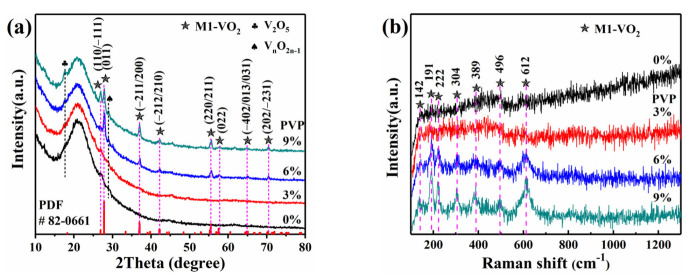
GIXRD pattern (**a**) and Raman spectra (**b**) of VO_2_ thin films prepared from VOCl_2_-PVP precursors with different PVP contents (0 wt.%, 3 wt.%, 6 wt.%, and 9 wt.%, respectively).

The phase composition of vanadium oxide films prepared by mixing different mass fractions of PVP in the precursor was determined from the glance incident XRD patterns and Raman spectra (Figure 1). As shown in Figure 1a, the broad peaks at 2*θ* of 15–25° were attributed to the amorphous quartz glass substrates. There were very weak diffraction peaks corresponding to the VO_2_ (M1) phase in sample P1 (0%) and sample P2 (3%) with low PVP content. As the content of PVP was increased to 6–9 wt.%, the peaks corresponding to the VO_2_ (M1) phase appeared, although very small amounts of impure phases of V_2_O_5_ and vanadium oxide Magnéli phases (V_n_O_2n−1_) emerged. The main peaks were indexed to the JCPDS Card No. 82-0661. It is well known that the chemical valence of V is variable due to the coexistence of many nonstoichiometric VO_2_ phases [33]. Nevertheless, this issue can be resolved by annealing in an appropriate concentration of NH_3_ and air [27,28]. However, the increase in PVP content seems to inhibit the preferred growth orientation of VO_2_ crystal on the (011) crystal plane, while diffraction peaks of other crystal planes go higher. The Raman scattering spectra (Figure 1b) of these four samples tested at room temperature were consistent with the XRD results. The tested Raman modes at 142 (B1g), 191 (Ag), 222 (Ag), 304 (Ag), 389 (Ag), 496 (Ag), and 612 (A1g) cm^−1^ were well marched with the pure VO_2_ (M1) phase [16,27,28,34]. With the increase in PVP content, it can be detected that the crystallization of the corresponding VO_2_ (M1) phase in the film improved. According to the results above, we can see that PVP is a good film-forming agent, which can form a network structure by complexing metal group VO^2+^ through crosslinking polymerization. The increasing content of PVP was helpful to improve the viscosity of the precursor sol. Under the same spinning speed, the VO_2_ film became thicker with the increase in the viscosity of precursor sol, resulting in the growth of VO_2_ crystals in certain lattice planes.

### 3.2. Microstructures of VO_2_ (M1) Thin Films

**Figure 2:**
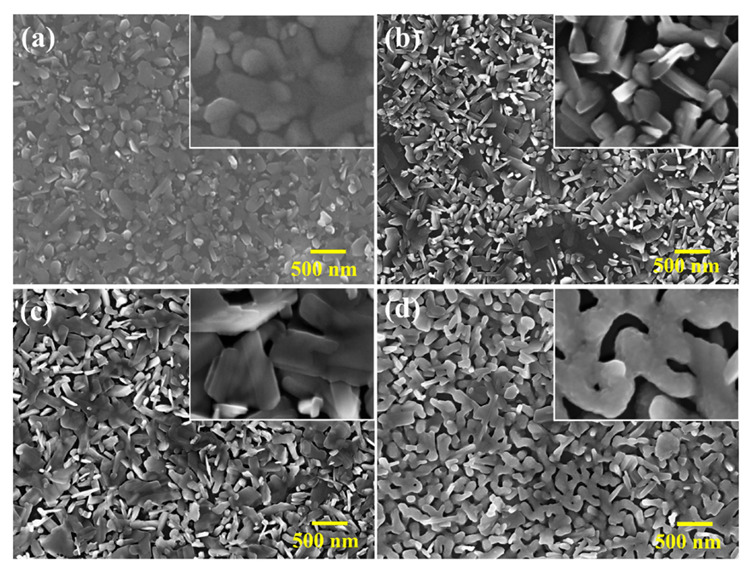
The top-view FESEM images (magnification = 20,000×; magnification for each inserted picture in the top right = 100,000×) of VO_2_ thin films prepared using different PVP contents in the precursor: 0 wt.% (**a**); 3 wt.% (**b**); 6 wt.% (**c**); 9 wt.% (**d**).

**Figure 3:**
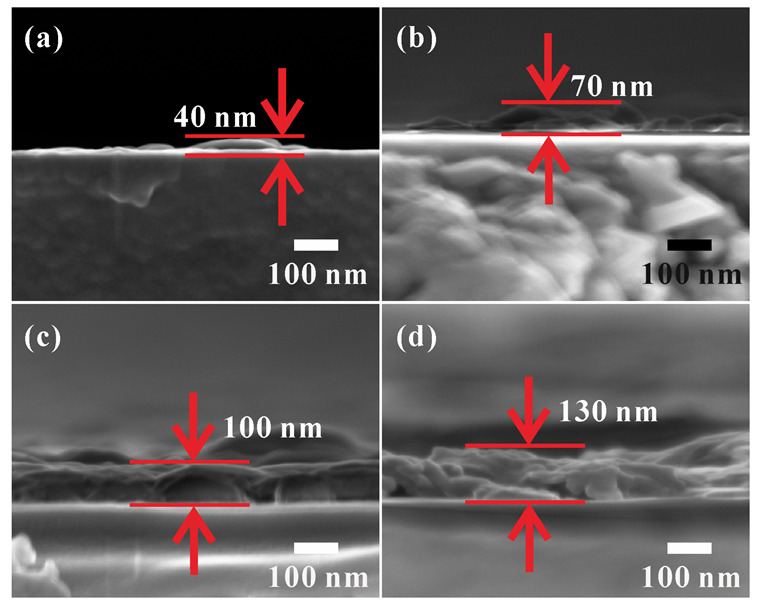
The cross-section photos of VO_2_ thin-film samples prepared using different PVP contents in the precursor: 0 wt.% (**a**); 3 wt.% (**b**); 6 wt.% (**c**); 9 wt.% (**d**).

The microstructure and thickness of VO_2_ films were observed from the top view and cross-sectional view by scanning electron microscopy (SEM), as shown in Figure 2 and Figure 3, respectively. Figure 2 displays that this group of film samples consisted of crystal sheets or tetragonal prism crystal particles with a large number of pores. Unlike the spherical or tetragonal morphology of the particles in [27,28], the crystals in Figure 2 exhibited short bar or prismatic shapes. All characteristic XRD peaks in Figure 1a were not very strong, indicating that the films were not well crystallized. Accordingly, we can see a disordered arrangement of crystal particles on the surface of the film. This result reveals that the change in the film sample morphology was closely related to the concentration of vanadium ion complexed with polymers in the precursor. The porosity of the films increased when the concentration of vanadium decreased. Due to the addition of PVP, the crystal particles in the films were packed from loose texture to tight arrangement. Crystal boundaries became obvious, consistent with the rise in peak intensity of some feature peaks of VO_2_ and V_2_O_5_ in Figure 1a. Furthermore, the crystal particle shape and size were more uniform, and the crystal angular morphology became smoother. Thus, a certain content of PVP could regulate the crystal particle morphology and the pore structure of VO_2_ films due to the space hindrance.

With the increase in PVP content, the crystal particles in the film sample became denser and more closely packed. The pores in the film reduced in number, and the crystal particle size distribution became more uniform. Otherwise, due to the increase in PVP content, the viscosity of the prepared sol increased. As a result, the thickness of the prepared film, as well as the adhesion strength of the film on the substrate, increased. As the film thicknesses could also be controlled by varying the spin-coating parameters, the spinning speed and coating times were fixed. The cross-section morphology of sample P1 prepared without PVP can be observed in Figure 3a, where only a small quantity of particles were attached to the glass substrate. The film formation of this sample was poor, and its adhesion to the substrate was weak in the absence of PVP. Most of the VO_2_ film was detached from the substrate during sample preparation. As can be seen from the cross-section, the film thickness of the sample increased from a few tens of nanometers (P2) to around 100 nm (P3), and the thickness of P4 increased to approximately 120–200 nm (Figure 3d). However, the edge and surface of the P4 film were rough, which could be ascribed to the increased viscosity of the precursor solution. On the one hand, the homogeneous deposition of sol on the substrate was hindered by the high viscosity; on the other hand, the crystalline process was hindered by the viscous resistance. Therefore, PVP from a high-viscosity precursor reduced the flatness of the VO_2_ film.

### 3.3. Chemical Analysis of VO_2_ (M1) Thin Films

**Figure 4:**
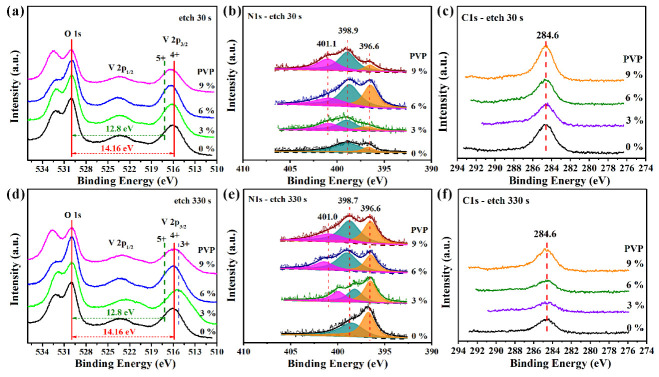
High-resolution XPS spectra of O *1s*–V *2p* (**a**,**d**), N *1s* (**b**,**e**), and C *1s* (**c**,**f**) core level energies for VO_2_ thin films prepared by different PVP contents in precursor: (**a**–**c**) after etching for 30 s by Ar^+^; (**d**–**f**) after etching for 330 s by Ar^+^.

The chemical composition of the vanadium oxide films was determined by XPS testing. To eliminate the effect of surface oxidation and contamination, a survey scan and high-resolution XPS measurements were performed after Ar^+^ ion etching. The XPS full-spectrum scan of the VO_2_ film sample showed that the film consisted of O, V, C, and N elements. Cl element appeared in the precursor gel volatilized by pyrolysis during the subsequent heat treatment. The chemical valence states of O *1s*, V *2p*, N *1s*, and C *1s* elements inside the film surfaces are shown in Figure 4. The corresponding etching depth was approximately 0.5 nm (Figure 4a–c) and 5.5 nm (Figure 4d–f) after Ar^+^ ion sputtering for 30 s and 330 s, respectively.

The V *2p*–O *1s* curves (Figure 4a,d) were calibrated according to the O *1s* core level at binding energies (*BE*) of 530.0 eV. The *ΔBE* values (the difference between O *1s* and V *2p_3/2_* core levels) for V^5+^, V^4+^, and V^3+^ were 12.8 eV, 14.16 eV, and 14.71 eV, respectively. Accordingly, the oxidation states of vanadium corresponding to V *2p* core level energies were V^5+^ (517.2 eV), V^4+^ (515.84 eV), and V^3+^ (515.29 eV) [35,36,37]. The V valence states inside the VO_2_ thin-film samples were V^4+^, while the V valence states near the film surface increased in valence to between V^4+^ and V^5+^. This indicates that the VO_2_ film exposed to air was gradually oxidized to V_2_O_5_ from the surface to the center. The bulges produced on the higher *BE* shoulder of O *1s* around 532 eV were due to the collective effects of satellite peaks of V^4+^ *2p*_1/2_, V^5+^ *2p*_3/2_, and V^3+^ *2p*_1/2_, as well as the influence of O–Si, O–H, O–C, or O–N bonds [37,38,39]. Si element was derived from the quartz substrate, while H, C, and N elements could have resulted from the pyrolysis of PVP and the NH_3_ atmosphere.

The C *1s* curves (Figure 4c,f) and N *1s* curves (Figure 4b,e) were calibrated according to the C *1s* core level at 284.6 eV. The C *1s* peaks of all samples located around 284.6 eV were mainly ascribed to the *sp*^2^ binding of the C–C bond, C–H bond, and C–O bond [40,41,42,43]. The C *1s* peak intensity of the film sample weakened with the etching of the Ar^+^ ion. Therefore, these detected C *1s* peaks may have been derived from the elemental carbon from the XPS instrument or the absorbed carbonate species from the environment. It is worth noting that the C atom was not doped into the VO_2_ crystal lattice, since no characteristic peak of the V–C bond (around 282 eV) was detected.

The XPS spectra of N *1s* peaks probed at depths of about 0.5 nm and 5.5 nm of VO_2_ thin-film samples are presented in Figure 4b,e, respectively. All broad N *1s* peaks could be resolved into two or three peaks with locations around 401 eV (molecularly adsorbed N_2_ or NO_x_) [44], 398.7–398.9 eV (N–H bond [27,45,46], N–O bond [47], or N–C bond) and 396.6 eV (V–N bond [27]). Thus, in addition to the N atoms doped into VO_2_ crystal lattice in the form of substitution, there were interstitial N atoms that could trap other nonmetallic atoms such as H, O, N, and C into voids of VO_2_ bulk phase through bonding. Compared with samples fabricated from PVP-containing precursors, the VO_2_ film sample (P1) prepared without PVP showed that the N source from the NH_3_ atmosphere mainly existed in the form of interstitial filling and substitutional doping. When PVP was added, adsorbed N species (N_2_ or NO_x_ molecules) were also detected in all film samples (P2, P3, and P4). Thus, the N introduced by PVP mainly appeared in the form of adsorption in VO_2_ film, while the N derived from the NH_3_ atmosphere promoted the substituted N-doping mode and formed V–N bonds as previously verified [27].

**Figure 5:**
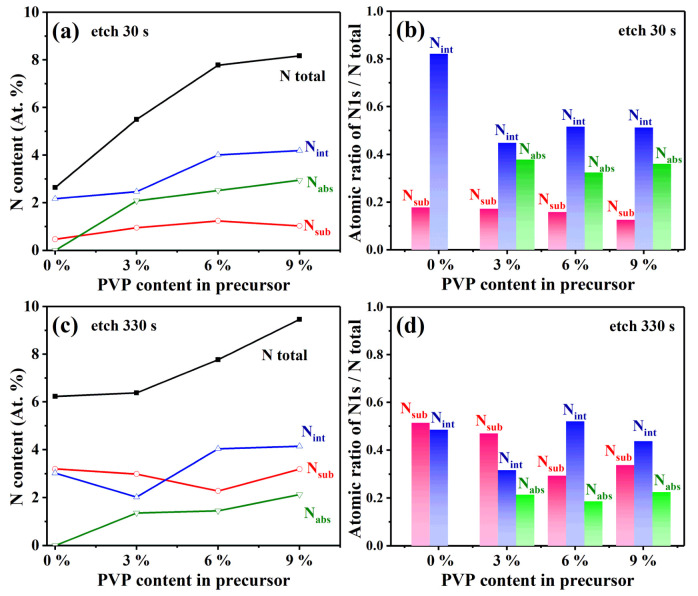
The content of N *1s* contained in N_sub_ (396.6 eV), N_int_ (399 eV), and N_ads_ (401 eV) (**a**,**c**), and the ratio of N *1s*/total N (**b**,**d**) in the VO_2_ thin films prepared using different PVP contents in precursor ((**a**,**b**): etching for 30 s, the corresponding etching depth was approximately 0.5 nm; ((**c**,**d**): etching for 330 s, the corresponding etching depth was approximately 5.5 nm).

The estimates of the concentration of N species in VO_2_ thin film could be extracted from the XPS data [48]. According to the different N *1s BE* locations, N *1s* peaks were divided into adsorbed N (N_ads_) around 401 eV, substituted N (N_sub_) close to 396.6 eV, and interstitial N (N_int_) around 399 eV. The concentrations of different N species inside VO_2_ thin-film samples are listed in Table 1. For further intuition, the correlations between the detected amount of nitrogen content in VO_2_ thin films and the PVP concentration in vanadium precursor are illustrated in Figure 5.

The contents of the N element and its different species in VO_2_ thin films are exhibited in Figure 5a,c. Both graphs show that the total N content and adsorbed N species (N_ads_) content gradually increased with the increase in PVP content in the vanadium precursor. As the etching time was prolonged, the probe depth increased, and the detected total N content was enhanced. In particular, because the V–N bond could be produced by Ar^+^ ion sputtering [49], the N_sub_ content (V–N bond) increased with the probe depth, while the variation of N_int_ content was not significant. Meanwhile, the content of N_ads_ in samples containing 3% to 9% PVP slightly decreased with the etching time. Furthermore, it is noticeable that the N_ads_ proportion with the same depth in VO_2_ thin film increased as the PVP content increased. Interestingly, this is the opposite of the influence rule of NH_3_ concentration on N_ads_ content in samples during heat treatment presented in a previous study [27].

The relative proportion of each N species in total N is illustrated in Figure 5b,d. It can be seen from the graphs that the increase in PVP from 3% to 9% content in the precursor did not affect the relative proportion of the three N species (N_ads_, N_sub,_ and N_int_) in thin-film samples. In the vicinity of the sample surfaces, all the N_sub_ proportions of prepared VO_2_ thin films are in the range of 10% to 20% whether the PVP was added or not. In comparison, the N_int_ proportion varied from 40% to 50%, and the N_ads_ proportion varied from 30% to 40% when the PVP was appended to the precursor. However, no N_ads_ species were detected in the sample without the addition of PVP in the precursor. Underneath the film surface, it was found that N_ads_ species, whose proportion was maintained around 20%, only existed in the samples with the addition of PVP in the precursor.

In summary, it was deduced from the VO_2_ film fabrication process that an N source could be introduced from pyrrolidone-N of PVP into a vanadium precursor. The N source originating from the NH_3_ atmosphere during heat treatment played different roles and led to different products. The NH_3_ atmosphere during heat treatment mainly played a role in promoting the substitution doping of nitrogen for oxygen in VO_2_. This is consistent with the conclusion verified in previous work [27]. The pyrrolidone-N may have introduced N-containing groups with N–H or N–C bonds into the vicinity of the surface or void of the film in the form of molecular adsorption or atom (N, O, H, and C) filling.

### 3.4. Optical Properties of N-Incorporated VO_2_ (M1) Thin Films

**Figure 6:**
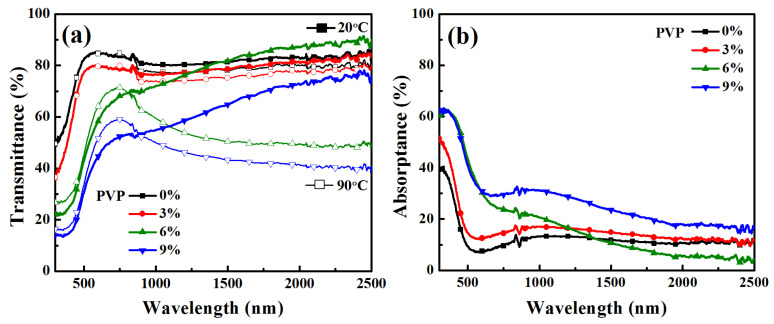
The UV/Vis/NIR transmittance (**a**) and absorptance (**b**) spectra of VO_2_ thin films prepared using different contents of PVP in precursor. The transmittance spectra were measured at 20 °C and 90 °C, respectively; the absorptance spectra were measured at 20 °C.

Figure 6 shows the optical transmission and absorption profiles of VO_2_ thin films prepared with different PVP contents in vanadium precursors. Table 2 illustrates the luminous transmittance (*T_lum_*), regulation efficiency of solar transmittance (*ΔT_sol_*), and modulation efficiency at 2000 nm (*ΔT_2000 nm_*) between ambient temperature (20 °C) and heated state (90 °C). It can be observed that the *T*_lum_ of the film decreased successively, while the values of *ΔT_sol_*, *ΔT_NIR_*, and *ΔT_2000 nm_* first increased with PVP concentration from 0 to 6 wt.%, and then decreased when the PVP concentration increased to 9 wt.%. The optical properties of different samples were affected by the phase composition and the thickness of the films, which varied with the content of PVP. Numerous well-crystallized VO_2_ (M1) crystal particles in films yielded a good thermochromic modulation effect, corresponding to high *ΔT_sol_* and *ΔT_NIR_*. However, the thick film was harmful to the transmittance of visible light, corresponding to lower *T_lum_*. Meanwhile, the increase in film thickness also led to the enhancement of light absorption by the film samples, as shown in Figure 6b. The optical absorption edges (λ*) [50] of VO_2_ thin films differed, resulting in the different colors of the films observed.

### 3.5. Band Structure of N-Incorporated VO_2_ (M1)

**Figure 7:**
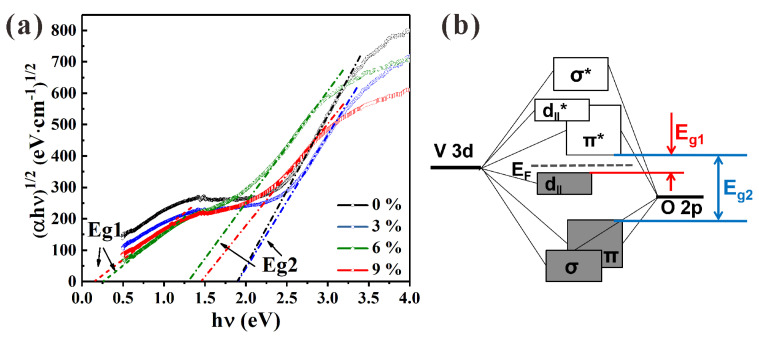
(**a**) Tauc plots of VO_2_ thin-film samples prepared using different contents (0, 3, 6, and 9 wt.%) of PVP in precursors. (**b**) Schematic illustration of band structure near the Fermi level for pure VO_2_ (M1).

The bandgap energy (*E_g_*) of N-incorporated vanadium oxide thin films at room temperature can be estimated from the Tauc formula [8,51,52]:

(1) $${\left(\alpha h\nu \right)}^{n}=A\left(h\nu -E_{g}\right),$$

where *hν* is the photon energy, *A* is a constant coefficient, and n was chosen to be 1/2 (most authors assume that M1 phase VO_2_ follows an indirect allowed transition). The optical absorption coefficients *α* can be obtained according to the following equation:

(2) α=1/d ln[(1−R)/T],

where *d* is the film thickness, *T* is the transmission, and *R* is the reflection. The transmission spectra and reflection spectra corresponding to 300–2500 nm could be measured directly, and the film thickness was averaged according to the observed sample cross-section. According to the Tauc plot results of samples P1 to P4 in Figure 7a, two kinds of different energy bandgaps were sketched.

According to octahedral crystal field theory, a bandgap exists between the antibongding *π* orbitals (π*) band at a higher level and the *d_ǁ_* band at a lower level in the vicinity of the Fermi energy level (*E*_F_) in the insulating phase VO_2_. This bandgap is called *E_g_*_1_. Another bandgap that corresponds to the bandgap between bonding *π* orbitals and π* is *E_g_*_2_. A schematic of the energy band structure of *E_g_*_1_ and *E_g_*_2_ is distinguished in Figure 7b [53,54,55]. *E_g_*_1_ is related to the MIT phase transition, whereas *E_g_*_2_ correlates with the visible-light transmittance and color.

For samples P1 and P2, only the energy gap *E_g_*_2_ appeared with the same value of approximately 1.90 eV. Both *E_g_*_1_ and *E_g_*_2_ were found in sample P3 and sample P4, of which *E_g_*_2_ increased from 1.31 to 1.45 eV. However, *E_g_*_1_ decreased from 0.26 eV to 0.16 eV with the increase in PVP content from 6 wt.% to 9 wt.%. Combining the changing trend of energy gaps in different PVP contents with the XPS results and optical performance, it could be conjectured that the adsorption of N species (N_ads_) on the surface and pores of the VO_2_ film and N species bonded with H, N, O, or C (N_int_) may have altered the values of *E_g_*_1_ and *E_g_*_2_, thereby regulating the phase transition of VO_2_ and the film color. However, the hypothesis of N adsorption and filling of elements such as H, N, O, or C inferred from XPS analysis on the surface and in the voids of the VO_2_ thin film needs further experimental verification.

**Figure 8:**
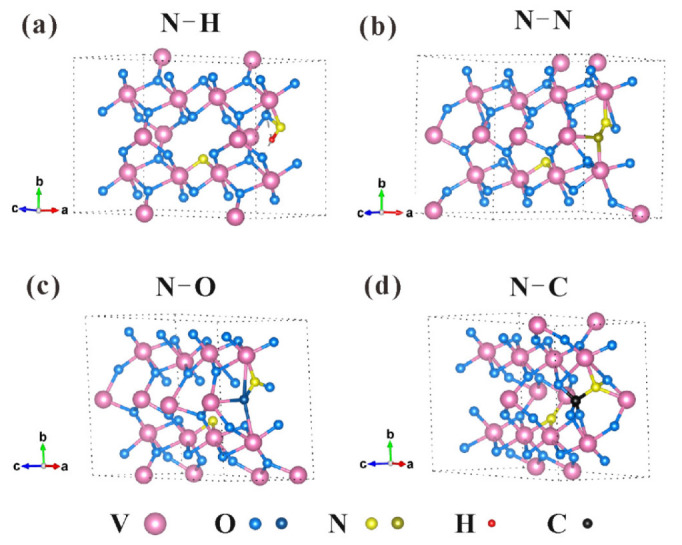
Calculation models of N-containing VO_2_(M1). (**a**) N_sub_, N_int_–H; (**b**) N_sub_, N_int_–N (N_ads_ of N_2_); (**c**) N_sub_, N_int_–O; (**d**) N_sub_, N_int_–C.

To reveal the possible fine-tuning effects of N species on optical properties (both *τ_c_* and color) of N-incorporated VO_2_ (M1) thin films, DFT calculations were performed using VASP. According to the XPS results of sample P4, the doping ratio of substitutional N atoms, interstitial N atoms, and adsorbent N relative to O atoms were all about 3%. Therefore, the chosen doping amount of each N species in the N-incorporated VO_2_ (M1) model established was about 3.13% (2.75 wt.%), in which one N atom was substituted for one of 32 O atoms. These calculations took substitutional N-doping, interstitial N-doping, and N-adsorption into account (Figure 8) to be in line with the experimental investigation. On the basis of previous calculation work [27] on pure VO_2_ (M1) and N-doped VO_2_ (M1), models of VO_2_ (M1) doped with 3.13% substitutional N atom, doped with 3.13% interstitial N atom, and bonded with H, N, O, or C atom were used. N_int_–N, N_int_–H, and N_int_–O could also be regarded as the adsorption of N_2_, NH_x_, and NO_x_, respectively.

**Figure 9:**
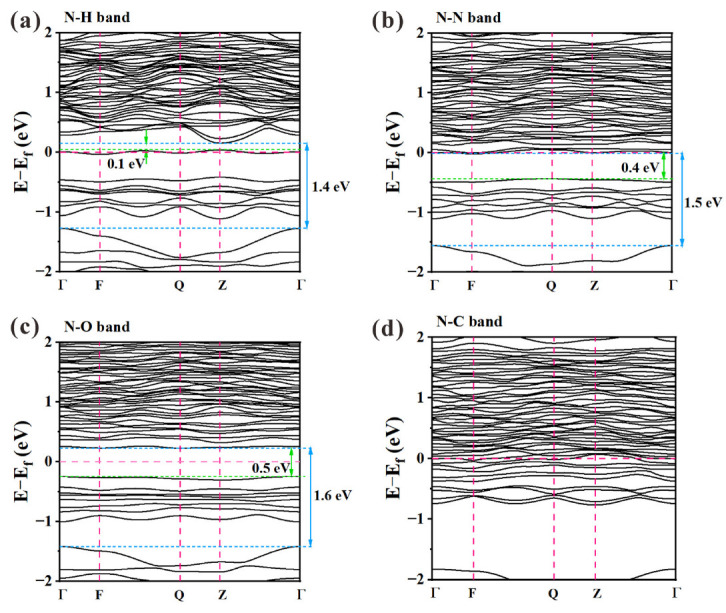
The calculated band structures of N-incorporated VO_2_ (M1) according to density functional theory (DFT) calculations. All calculations were based on the corresponding models in Figure 8.

The simulated band structures for the N-doped VO_2_ (M1) absorbing H, N, O, and C atoms are illustrated in Figure 9. The various energy levels of N-containing VO_2_ (M1) calculated from Figure 9 are summarized in Table 3. Concerning previous work [27] for pure VO_2_ (M1), *E_g_*_1_ was taken as 0.62 eV, and *E_g_*_2_ was taken as 1.44 eV. Considering the combined effect of N_sub_ and N_int_ with additional atoms (C, H, O, or N), a comparison of calculation results and estimated experimental results is also provided in Table 3. The calculated *E_g_*_1_ values of N-incorporated VO_2_ (M1) ranged from 0.1 eV to 0.5 eV, and the values of *E_g_*_2_ ranged from 1.4 eV to 1.6 eV. The theoretical calculation results of *E_g_*_1_ were generally smaller than those of pure VO_2_ (M1), indicating that the introduction of N, including doping and adsorption, decreased the phase transition temperature (***τ_c_***) of VO_2_. Furthermore, the existence of N_int_–H could sharply decrease the *E*_g1_, in good agreement with the absence of *E*_g1_ in P1 and P2 samples. Similarly, the appearance of *E*_g1_ in P3 and P4 could be attributed to the existence of N–N (N_2_ molecule adsorption) and N–O bonds. Unlike the N–H, N–N, and N–O cases in N-incorporated VO_2_ (M1), the band structure of the case containing the N–C bond showed metal properties. The slight increase in *E_g_*_2_ could have theoretically caused a decrease in the near-infrared absorption of VO_2_, thereby enhancing the *T_lum_* of VO_2_ film and diluting the film color. However, in contrast to the theoretical calculation results, the experimental values of *E_g_*_2_ varied from 1.31 eV to 1.90 eV, which could have been mainly affected by the crystal phase composition and film thickness.

## 4. Conclusions

N-incorporated VO_2_ (M1) thin films were synthesized through a PVP-assisted sol–gel route followed by heat treatment in vacuum containing a small amount of NH_3_ gas. The microporous structure, crystallization, film thickness, and optical properties of VO_2_ (M1) film could be effectively regulated by varying the PVP concentration in the vanadium precursor solution. XPS analysis demonstrated that the N element introduced by the pyrrolidone-N in PVP mainly existed in the form of N_2_ or NO_x_ molecular adsorption in VO_2_ film or combined atom (N, O, and H) filling in the vicinity of the surface or void of the film. The subsequent annealing in NH_3_ was attributed to the generation of substitutional N–V bonds in the VO_2_ crystals. As the PVP concentration increased, *T_lum_* of the VO_2_ thin film decreased due to the increase in film thickness, while *ΔT_sol_* and *ΔT_NIR_* first increased and then decreased. The best results of thermochromic film in this work showed a *T_lum_* of 52.19–56.79%, a *ΔT_sol_* of 4.99%, a *ΔT_NIR_* of 7.39%, and a *ΔT_2000 nm_* of 37.6%. Both the Tauc plot estimation and the theoretical calculation suggested that the combined effect of substitutional N atom, interstitial N–X species (X = H, N, or O), and adsorbed N-containing molecules decreased the *τ_c_* due to the reduction in *Eg*_1_. According to the calculated value of *Eg*_2_, the introduction of elements such as N, H, or O had little effect on the bandgap between π and π* in the insulating VO_2_ (M1) phase. Actually, the film thickness and the micro-morphology had significant impacts on the visible-light transmittance and color of the films. This work presents an approach to adjust the optical properties of VO_2_ (M1) film, which is expected to be applied to smart windows or other VO_2_-based devices.

## Figures and Tables

**Table 1:** The concentration of different N species in VO_2_ thin films, as determined by XPS analysis after Ar^+^ sputtering for 30 s and 330 s.

Sample	PVP Content (wt.%)	N/(N+O)	N_sub_	N_int_	N_ads_	N/(N+O)	N_sub_	N_int_	N_ads_
		Etching for 30 s				Etching for 330 s			
P1	0	2.64%	0.47%	2.17%	0	6.23%	3.20%	3.03%	0
P2	3	5.50%	0.95%	2.47%	2.08%	6.37%	2.99%	2.02%	1.36%
P3	6	7.78%	1.24%	4.01%	2.52%	7.77%	2.28%	4.04%	1.45%
P4	9	8.17%	1.03%	4.19%	2.95%	9.46%	3.19%	4.14%	2.13%

**Table 2:** Optical properties of VO_2_ thin films prepared using different contents of PVP in the precursor.

Sample	PVP Dosage (wt.%)	Film Color	λ* (nm)	*T_lum_* (%)		*∆T_sol_* (%)	*∆T_NIR_* (%)	*∆T_2000 nm_* (%)
				20 °C	90 °C			
P1	0	Pale yellow	426	84.08	84.03	0.92	1.02	2.9
P2	3	Pale yellow	432	78.95	78.95	0.97	1.03	3.9
P3	6	Yellow green	472	52.19	56.79	4.99	7.39	37.6
P4	9	Yellow green	482	39.26	44.55	1.27	4.17	31.3

**Table 3:** Calculated energy levels (in eV) of VO_2_ and N-containing VO_2_ (M1) in contrast with previous calculations [27] and experimental results.

	Pure VO_2 cal._	N: VO_2_ (N-sub-int-ads) _cal._				N: VO_2 exp._			
		N–H	N–N	N–O	N–C	P1	P2	P3	P4
*Eg_1_*	0.62	0.1	0.4	0.5	-	-	-	0.26	0.16
*Eg_2_*	1.44	1.4	1.5	1.6	-	1.90	1.90	1.31	1.45

## Data Availability

Not applicable.

## Abbreviations

Nomenclature for the symbols and abbreviations

VO_2_ (M1):	M1 phase VO_2_
PVP:	polyvinyl pyrrolidone
*T_lum_*:	visible integrated transmittance
*T_sol_*:	solar integrated transmittance
*ΔT_sol_*:	solar modulation efficiency
*ΔT_NIR_*:	near-infrared modulation efficiency
*ΔT_2000 nm_*:	modulation efficiency at 2000 nm
*τ_c_*:	critical temperature of phase transition
*E*_F_:	Fermi energy level
*E_g_*:	bandgap energy
*E_g1_*:	bandgap energy related to metal-to-insulator transition
*E_g2_*:	bandgap energy related to the visible transparency
GIXRD:	glance incident X-ray diffraction
XPS:	X-ray photoemission spectroscopy
FESEM:	field-emission scanning electron microscopy
UV/Vis/NIR:	ultraviolet/visible/near-infrared light
DFT:	density functional theory
VASP:	Vienna Ab initio Simulation Package
N_ads_:	adsorbed N
N_sub_:	substituted N
N_int_:	interstitial N
